# Reimagining Healthcare: Unleashing the Power of Artificial Intelligence in Medicine

**DOI:** 10.7759/cureus.44658

**Published:** 2023-09-04

**Authors:** Javed Iqbal, Diana Carolina Cortés Jaimes, Pallavi Makineni, Sachin Subramani, Sarah Hemaida, Thanmai Reddy Thugu, Amna Naveed Butt, Jarin Tasnim Sikto, Pareena Kaur, Muhammad Ali Lak, Monisha Augustine, Roheen Shahzad, Mustafa Arain

**Affiliations:** 1 Neurosurgery, Mayo Hospital, Lahore, PAK; 2 Epidemiology, Universidad Autónoma de Bucaramanga, Bucaramanga, COL; 3 Medicine, Pontificia Universidad Javeriana, Bogotá, COL; 4 Medicine, All India Institute of Medical Sciences, Bhubaneswar, Bhubaneswar, IND; 5 Medicine and Surgery, Employees’ State Insurance Corporation (ESIC) Medical College, Gulbarga, IND; 6 Internal Medicine, Istanbul Okan University, Istanbul, TUR; 7 Internal Medicine, Sri Padmavathi Medical College for Women, Sri Venkateswara Institute of Medical Sciences (SVIMS), Tirupati, IND; 8 Medicine/Internal Medicine, Allama Iqbal Medical College, Lahore, PAK; 9 Medicine, Jahurul Islam Medical College, Kishoreganj, BGD; 10 Medicine, Punjab Institute of Medical Sciences, Jalandhar, IND; 11 Internal Medicine, Combined Military Hospital, Lahore, PAK; 12 Medicine, Kasturba Medical College, Manipal, Manipal, IND; 13 Medicine, Combined Military Hospital (CMH) Lahore Medical College and Institute of Dentistry, Lahore, PAK; 14 Internal Medicine, Civil Hospital Karachi, Karachi, PAK

**Keywords:** new insights, modern medicine, newer technology in healthcare, artificial intelligence in healthcare, ai & robotics in healthcare

## Abstract

Artificial intelligence (AI) has opened new medical avenues and revolutionized diagnostic and therapeutic practices, allowing healthcare providers to overcome significant challenges associated with cost, disease management, accessibility, and treatment optimization. Prominent AI technologies such as machine learning (ML) and deep learning (DL) have immensely influenced diagnostics, patient monitoring, novel pharmaceutical discoveries, drug development, and telemedicine. Significant innovations and improvements in disease identification and early intervention have been made using AI-generated algorithms for clinical decision support systems and disease prediction models. AI has remarkably impacted clinical drug trials by amplifying research into drug efficacy, adverse events, and candidate molecular design. AI’s precision and analysis regarding patients’ genetic, environmental, and lifestyle factors have led to individualized treatment strategies. During the COVID-19 pandemic, AI-assisted telemedicine set a precedent for remote healthcare delivery and patient follow-up. Moreover, AI-generated applications and wearable devices have allowed ambulatory monitoring of vital signs. However, apart from being immensely transformative, AI’s contribution to healthcare is subject to ethical and regulatory concerns. AI-backed data protection and algorithm transparency should be strictly adherent to ethical principles. Vigorous governance frameworks should be in place before incorporating AI in mental health interventions through AI-operated chatbots, medical education enhancements, and virtual reality-based training. The role of AI in medical decision-making has certain limitations, necessitating the importance of hands-on experience. Therefore, reaching an optimal balance between AI’s capabilities and ethical considerations to ensure impartial and neutral performance in healthcare applications is crucial. This narrative review focuses on AI’s impact on healthcare and the importance of ethical and balanced incorporation to make use of its full potential.

## Introduction and background

In the modern world, the doctor-patient encounter has rapidly risen due to the introduction of artificial intelligence (AI). The cost of healthcare and the uneasy accessibility form the primary issues when it comes to the availability of healthcare in rural and urban areas. Besides, the number of treatment providers and the inability to cope with rising technology is also becoming a major problem. Therefore, to cope with the rising spread of disease and to be able to provide a more budget-friendly and timely cure, AI is the next best step [1].

The tiresome task of drug development and medicine manufacture, which required heavy investments and human intervention earlier, has now been reduced to a click. Ranging from cure identification to execution of its use has all become an AI-monitored task. Hence, effectiveness and efficiency are considerably targeted when the role of machines and new technology comes into play [2].

With accumulated laboratory technological data combined with analysis of patient history, AI proved to be nothing short of a blessing during the COVID-19 pandemic. A complete review of the post-COVID-19 syndromes helped to improve vaccine development. Thus, AI is a multifaceted approach to not just curative but also preventive measures [2].

While there is no doubt that AI has revolutionized healthcare to its core and is continuing to do so, ethical and private concerns form a major part of the demerits associated with it. The first problem is the unavailability of credible data. The second problem is algorithm dysfunction. Safe storage of data and elimination of breach of information forms the basis of patient privacy. Ethically, genomic replication or even human clone formation is resented by many cultures and religions worldwide [3]. Therefore, this paper provides insights into personalized treatment and precision medicine, drug discovery, and how AI has made its way into several domains of society. From disease management to patient monitoring, AI forms the backbone of advanced medicine [4]. Therefore, this paper focuses on answering the question, “How can AI change the future of a standard patient-physician encounter while also optimizing procedures and diagnostics aspect of it?” [1].

## Review

Selection process

We defined the criteria for selecting articles, including publication date, relevance to the topic, study design, and geographic scope, and used multiple databases (PubMed, Google Scholar, Web of Science, etc.) and keywords to search for articles related to the topic and to ensure we capture all relevant studies. Subsequently, the authors reviewed titles, abstracts, and full texts to identify potential articles meeting inclusion criteria and excluded those that did not meet the criteria. We also checked reference lists of selected articles to find additional relevant sources that may have been missed initially. Some authors evaluated the quality of selected articles based on factors such as study design, methodology, sample size, and author credibility. Finally, we extracted the key information from each selected article, including findings, methodologies, and conclusions, and organized this information systematically.

Artificial intelligence-driven diagnostics and imaging

AI employs algorithms such as machine learning (ML) and deep learning (DL) to diagnose diseases in a time-efficient manner. This helps diagnose diseases early and reduces complications [5]. A clinical decision support system (CDS) aids clinicians in diagnosing and treating diseases. CDSs such as diagnostic CDS aid in diagnosing using rules generated automatically or manually. This has been successful, as it has been tested in electronic medical records (EMRs) [6]. Newer DL methods have been used to construct contrast images utilizing the details from non-contrast imaging. A two-dimensional cycle generative adversarial network (cycleGAN) was used for this reconstruction. Development and improvement in this model can avoid the complications associated with contrast use [7].

Time is crucial for the early identification and containment of epidemics. AI-based deep Q learning auxiliary diagnosis (DQAD) was used to diagnose epidemics, reducing the time of diagnosis compared to the current methods [8]. Using chest computed tomography (CT) imaging, a deep learning model called COVID-19 detection neural network (COVNet) was built to correctly identify COVID-19 from community-acquired pneumonia and other lung conditions [9].

A highly efficient shock advice algorithm (SAA) is essential for an automated external defibrillator to diagnose and function correctly. A new SAA with DL and ML algorithms had better sensitivities and specificities than the existing SAA. Researchers used magnetic resonance imaging (MRI) histogram peaks to design AI algorithms that accurately detect tumor volumes, with improved specificity, sensitivity, and interoperator repeatability. ML models combining radiomics and clinical indicators have successfully predicted molecular subgroups of medulloblastomas and differentiated skull base chondromas from chondrosarcomas [10].

Diagnosing stroke early is essential as a delay in endovascular thrombectomy (EVT) reduces effectiveness. MethinksLVO (large vessel obstruction), an ML software, uses non-contrast computed tomography (NCCT) to identify the LVO location to facilitate the transfer of patients to EVT facilities early, thus improving outcomes [11]. The use of fully convolutional neural networks (fCNN) in reconstructing a volumetric brain from CT head films has proven to be a time-saving application [12]. AI-driven applications are used on smartphones to track health-related information and diagnose different conditions [13]. Kardia mobile cardiac monitor (KMCM) used an AI algorithm to detect heart rhythm as normal, possible Afib, and unclassified. A study putting this to the test against 12-lead electrocardiography (ECG) found that the KMCM had similar sensitivities and specificities compared to the 12-lead ECG. For unclassified rhythm, the sensitivities and specificities increase if the KMCM rhythm is interpreted by a physician [14].

The role of AI in diagnosing certain types of cancers, such as pancreatic cancer, which is often diagnosed in late stages, could be life-changing for many patients. Many challenges occur in the diagnosis, such as late symptom onset, inconsistent symptoms, and lack of molecular markers. AI tools such as K-nearest neighbor (KNN), artificial neural network (ANN), and support vector machine (SVM) are studied to identify subtle abnormal imaging findings that aid in the early detection of pancreatic cancer [15]. The AI-powered ML algorithm was used to predict the probability of developing lung cancer after initial screening with the low-dose CT scan. It matched the accuracy of predicting with the Brock scoring system, but the AI-based system was better if the radiomic scores were extreme [16]. In breast cancer (BC) screening, lesion identification and classification are the two basic processes AI offers. Early CNNs are used to identify breast lesions. Whereas deep CNN, a DL algorithm, helps identify and classify lesions by studying the images [17].

Biopsy remains the gold standard method for diagnosing skin cancers. Using DL algorithms on fluorescence spectroscopy, basal cell carcinoma (BCC) was diagnosed in real time. DSL-1 was the device employed, which used the fluorescence spectra data from BCC lesions and normal skin as a base to identify the lesions [18]. A study was done to compare the CNN, a DL algorithm, and dermatologists’ diagnosis of melanoma and found that the former achieved higher sensitivities and specificities in diagnosing melanoma and nevi. It concluded that these algorithms could assist dermatologists in diagnosing melanoma given further studies [19].

Tumor borders that are difficult to identify become challenging during surgery, such as gliomas, depending on frozen sections for delineating the boundaries in real time but at the cost of time. Fluorescent light CNN (FLCNN) was studied using the near-infrared window (NIR-II) images as an alternative or in conjunction with the surgeon’s optical judgment. The study showed that the FLCNN captured the details of the image better than the surgeon [20].

AI’s role in diagnosing neurological diseases is being explored further [21]. Battineni et al. studied four different ML algorithms, namely, naïve Bayes (NB), ANN, KNN, and SVM, individually and in combination to predict Alzheimer’s disease (AD). The study concluded that the combined models, with limited features, predict AD at the initial stages with greater accuracy [22]. Various ML and DL algorithms are studied for their use in diagnosing other neurological diseases such as Parkinson’s disease (PD), major depressive disorder (MDD), attention-deficit hyperactive disorder (ADHD), autism spectrum disorder (ASD), and psychiatric disorders such as schizophrenia [23].

Currently, the diagnosis of obstructive sleep apnea (OSA) is made by polysomnography (PSG) which employs the apnea-hypopnea index (AHI). AI-based CNN algorithm uses a single-lead ECG signal to diagnose OSA with reasonable accuracy, sensitivity, and specificity. This method is easy and efficient but still needs PSG to confirm if the estimated risk is high [24]. AI’s role in diabetes care is to ease the aspect of monitoring. The self-monitoring system uses AI to detect hypoglycemia one hour prior and notifies the patient. AI is also used in retinal screening, diabetes diagnosis support, and risk categorization [25].

The use of AI in diagnosing hematologic disease is on the rise. Cellavision, a peripheral blood smear analyzer approved by the Food and Drug Administration (FDA), utilizes the ML-ANN algorithm to perform tests such as complete blood count, cell differential, identification, and location [26]. Morphogo, a CNN-based bone marrow analyzer, identifies and classifies hematopoietic tumor cells. It also identifies non-hematopoietic metastatic cells with greater accuracy and specificity [27]. Other systems such as Scopio, Mantiscope, and Vision Hema use different AI algorithms to diagnose hematologic conditions [28]. Cytogenetics remains the primary diagnostic testing for genetic disorders, including malignancies. A new AI-based tool called chromoEnhancer uses Generative adversarial networks (GAN) to produce good-quality karyograms. This helps detect the abnormality efficiently [29].

AI-driven diagnostics and imaging are proliferating and finding applications in every possible field of medicine. Further exploration into these algorithms can be assimilated to provide better patient care, considering the ethics and social regulations [30].

Personalized treatment and precision medicine

Artificial Intelligence in Genomics and Its Role in Personalized Treatment Plans

Personalized medicine is a method that considers an individual’s unique genetic makeup, environment, and lifestyle for treating and preventing diseases [31]. It shifts away from traditional diagnostic methods that rely on shared patient characteristics, aiming to transform healthcare through prevention, personalization, and precision [32]. To achieve this, various data sources such as genomics, biological data, transcriptomics, and proteomics play a crucial role [33]. Big data is essential in this process, and researchers are increasingly exploring the potential of AI for robust data analysis [34,35]. These advanced tools empower us to identify precision intervention targets and personalized medical approaches for each patient [36].

ML is a method that employs mathematical algorithms to construct models using datasets; these models can enhance their performance through experience [35]. Among the various ML techniques, DL utilizes neural networks, allowing end-to-end learning directly from raw data and seamless integration of different data types [37].

Functional genomics explores the intricate interplay between genetic attributes and environmental conditions. This endeavor has been significantly aided by the utilization of advanced deep architectures [37]. For instance, a notable example is DeepBind, a fully autonomous standalone software designed to predict the sequence specificities of DNA and RNA binding proteins [38]. Another noteworthy tool, DeepSea (a DL-based sequence analyzer), employs DL techniques to forecast the chromatin effects of sequence alterations at the single-nucleotide level. By learning from extensive chromatin profiling data, DeepSea effectively identifies the functional implications of non-coding variants. It is worth noting that the majority of disease-associated single-nucleotide polymorphisms (SNPs) reside within the non-coding regions of the genome. Additionally, the groundbreaking DeepMind AlphaFold method has revolutionized protein structure prediction, fundamentally altering a once-daunting challenge into a resolved matter [39,40].

Significant strides have been made in applying functional genomics to enhance precision medicine for prevalent non-cancerous conditions, such as kidney disease [32]. An example of an ML application using tissues, Liu et al. used *in silico nano dissection*, an ML algorithm, to study mRNA expression in glomeruli from IgA nephropathy patients [41]. They identified cell-specific genes differentially expressed compared to healthy controls. In cancer, AI applied to genomics, some classification models have been developed to help stratify individuals into high-risk and low-risk groups. Vural et al. implemented an unsupervised clustering approach to identify subgroups based on individual omics systems; the resulting model successfully identified three groups of breast cancer patients and associated specific genes with disease progression [42]. In another study by He et al., researchers focused on the identification of cancer-selective combinatorial therapies for ovarian cancer using supervised ML algorithms. They analyzed genomic and expression data from ovarian cancer patients and pan-cancer markers to predict drug targets and mutations in the study to identify top-ranked drug combinations with high accuracy, offering potential therapeutic options for personalized treatment [43].

ML models have the potential to analyze multimodal data obtained from electronic medical records and other curated sources to identify patients who could benefit from early treatment or participation in randomized control trials of innovative interventions [44]. For instance, in a study by Dong et al., extensive pharmacogenomics analyses were conducted across more than 1,000 cell lines. The aim was to gain insights into the mechanisms underlying the responses of anticancer drugs. In this research, a sophisticated SVM model was developed and evaluated, utilizing genomic data to accurately predict the sensitivity of anticancer drugs [45].

Drug Discovery and Development With Artificial Intelligence Assistance

AI is already deeply embedded in almost every facet of modern society. In exchange, it is undergoing rapid innovation. Because of this, it is rapidly gaining importance in the pharmaceutical industry, particularly in the realm of drug research and development [46].

Drug development is time-consuming and labor-intensive, predicated on iterative trial-and-error experimentation and high-throughput screening processes. On the other hand, using algorithms such as ML and natural language processing (NLP) will significantly speed up the entire process while also requiring substantial data inputs. Errors may be reduced, and the quality of analytical findings improved by streamlining the process [47].

Using the DL method makes it easier to ascertain the effectiveness of a substance. Using AI techniques, we can also determine if a medicine is toxic [48]. How AI may generate and synthesize novel molecules has been studied, opening the door to using AI to create new medicines [49]. Nonetheless, there may be ethical considerations related to medicine that limit its use [50].

AI makes use of a number of methods, including reasoning, knowledge representation, problem resolution, and a model of ML. This analysis will focus on the SVM classification model. SVM is a supervised machine learning module that generates analytical outputs by classifying two distinct sets of troublesome subgroups using a hyperplane between their extreme edges. Therefore, with the use of a decision boundary or hyperplane, the two sets can be organized into usable data [51].

ANNs are also used in the domain of ML known as DL. These can be described as a collection of high-tech computers that mimic the way in which the human brain sends and receives electrical signals by using *perceptons* that are analogous to biological neurons. An ANN is a collection of nodes that accept various inputs and convert them to the output, either individually or in a multi-link configuration, using algorithms to solve problems [52].

Anticipating global infectious disease hazards using AI-based surveillance models may be very helpful [53,54]. The key to building an effective surveillance system is to use an integrated modeling method that incorporates many kinds of individual data models, such as travel, cell phone location tracking, epidemiology, and behavioral pattern data [55]. A single person’s level and, more significantly, mass collecting awareness of the intended population is essential for this modeling technique to effectively control the transmission of infection [56]. Possible applications for such an integrated model-based platform include anticipatory surveillance and the identification of hazards posed by infectious illnesses of global importance. The use of mathematical models that can assess the transmission of infectious illnesses in mass gatherings and simulate the impact of public health measures at both the national and international levels is also encouraged by such integrated techniques [57].

Artificial intelligence-enabled patient monitoring and disease management

Public health surveillance faces data sourcing and analytics challenges in identifying reliable signals of health anomalies and disease outbreaks from data sources. AI helps tackle these challenges by enabling and enhancing public health surveillance through methods and techniques such as DL, reinforcement learning, knowledge graphs, Bayesian networks, and multiagent systems. AI enhances surveillance of various open novel or unexplored data, unstructured and semi-structured data, complex spatiotemporal data, and epidemics’ evolution, which is difficult with traditional surveillance methods [55].

Remote patient monitoring (RPM) or telemonitoring uses digitally transmitted health-related data to improve patient care through education, early disease decompensation detection, intervention, and enhanced patient-physician relationship [58]. Telemonitoring technology uses digital health platforms to enable patient evaluation outside a typical clinical visit [59]. A meta-analysis found that type 2 diabetes patients followed through mobile technologies for RPM and clinical advice delivery had improvement in glycemic control by 0.8% compared to standard care [60]. The application of AI in RPM includes monitoring vital signs, physical activities, diabetes, and mental health. AI can build patient-specific models using ML techniques for patients in the emergency department and ICU [61]. In a retrospective study by Taylor et al., a local big data-driven ML approach had better in-hospital ED patient mortality than existing clinical decision rules and traditional analytical techniques [62].

An ML approach predicted cardiac arrest within 24 hours more accurately than the traditional modified early warning scores for critically ill patients in the emergency department [61]. A retrospective cohort study by Antunes et al. showed that the new chronic liver failure Consortium Acute Decompensation Score (CLIF-C ADs) machine has statistically significant superiority in predicting 30-day mortality over traditional models [63]. RPM is being increasingly adopted following the COVID-19 pandemic [59].

Vipin et al. proposed an edge AI-enabled Internet of Things (IoT) healthcare monitoring system for the real-time scheduling of patients and provision of resources on a priority basis depending on the patient’s condition. This tool collects and transmits data and triggers appropriate action on other integrated devices, simplifying the process of monitoring and assistance provision, which is particularly useful in the elderly or disabled, and in pandemic situations [64].

The FDA issued Emergency Use Authorization for wearable and mobile ECG technologies to record QT intervals in patients taking hydroxychloroquine or azithromycin. However, AI-ECG technologies need further evaluation for external validity in diverse populations to be applied in routine clinical practice [65]. AI-based surveillance can be used to identify and disperse need-based support. However, unequal representation and opportunities can lead to malfunctioning and unpredicted health risks [66]. For example, biased distribution of health surveillance technology between countries can impact the efficacy of the tools, resulting in unfairness [67]. Potential biases can be introduced willingly or unconsciously. For instance, including objective perceptions and opinions can lead to bias in the incorporation of data and can affect the conclusions [68]. Algorithmic design development with the right fairness for a context poses a challenge [69]. An example is disparities in the distribution and access to COVID-19 vaccines in the United States due to disharmonized priority settings in the algorithm [70]. Data privacy and security, transparency, fairness of the analytical AI models, and limited computing power of sensing processors are a few other technical and regulatory challenges with wearable sensors with AI-at-the-edge functionality [71]. Phenotypic personalized medicine (PPM) aims to provide a quantitative representation of an individual patient’s overall health, while directly translating this knowledge into clinical care helps to find a suitable drug combination quadratic phenotypic optimization platform (QPOP) and dosing (CURATE.AI) based on data from previously treated patients. PPM-based approaches have produced superior results with limited side effects and maximized desired output than standard care [72]. Applications empowered by AI hold the capacity to assist in devising treatment plans for individuals with valvular heart conditions. AI-driven clinical decision systems enhance procedure planning of aortic valve replacement by integrating anatomical data, aiding valve size/type determination with speed and accuracy. It is beneficial for high-volume sites where manual imaging analysis is time-consuming and variable [73,74].

Artificial intelligence for remote and telemedicine

Telemedicine refers to delivering healthcare services using information and communications technologies, considering distance and accessibility as critical factors. Initially envisioned for tech-savvy patients, the COVID-19 pandemic propelled telemedicine into mainstream healthcare globally [75]. Its scalability enabled essential health and safety protocols during the crisis and established it as a sustainable approach beyond the pandemic. DL is favored due to its capacity for autonomous learning and adaptation [75]. AI algorithms have been extensively studied and implemented in various medical fields to streamline exam interpretations, enhance diagnostic accuracy, and reduce time and workforce requirements [76]. In modern medicine, patient data includes binary and numerical information and vast image data. Traditional computer programs often struggle to interpret such detailed reports. However, modern AI has demonstrated its efficiency in processing and analyzing complex image data [76].

A typical deep neural network utilized for processing and analyzing visual images is the CNN [76]. This network comprises multiple processing layers, allowing it to extract meaningful patterns and features from visual data [77]. In ophthalmology, diabetic retinopathy (DR) is a common complication of diabetes mellitus (DM) and a leading cause of preventable blindness [77]. The World Health Organization (WHO) recommends annual screening with dilated funduscopy for DM patients to prevent vision loss [78]. However, the significant number of DR patients poses challenges regarding screening, timely referral, and treatment due to the availability of human assessors and long-term financial sustainability. As a solution, developing DL and digital technology is advocated to facilitate the DR management process [76].

Similarly, age-related macular degeneration (AMD) is a significant cause of vision loss among the elderly population globally. However, the growing number of AMD patients and frequent follow-ups create a pressing need for a robust automatic mechanism. DL and telemedicine offer potential solutions for AMD management, including initial screening, subsequent monitoring, and treatment prediction. DL algorithms can assess visual acuity (VA) and optical coherence tomography (OCT) findings, aiding in determining AMD treatment strategies [79].

In the mental health domain, a smartphone-sensing wearable device was proposed to assess behavior and identify depressive and manic states in patients with bipolar disorder [80]. Patient monitoring is also advanced using AI [75]. In 2016, a research project focused on detecting fetal ECG signals and other physiological data of a fetus to enhance the monitoring and identification of fetal conditions during pregnancy [81]. Similarly, in 2017, the introduction of Wi-Mon, a wireless continuous patient monitoring system, addressed the need for ongoing patient monitoring by employing the Wireless Body Area Network (WBAN) concept, particularly for individuals requiring it, such as dengue patients [82]. Furthermore, a mobile cyber-physical system for healthcare is recommended using wearable devices to enable continuous patient monitoring and provide real-time updates to doctors or family members on the patient’s status [83].

Telemedicine, especially in dermatology, has rapidly embraced AI due to increasing demand, the necessity for high-quality images, and the availability of advanced technology [84]. While in-person diagnosis remains more accurate than teledermatology, telemedicine offers the potential to enhance dermatological care by providing convenience, reduced waiting times, and diagnostic support during case reviews [85]. In face-to-face assessments, AI delivers real-time diagnostic support, significantly enhancing dermatologists’ accuracy in identifying skin disorders [86]. For non-dermatologists, who may have varying expertise in skin conditions, AI-assisted diagnosis can be particularly valuable, especially in cases such as diagnosing melanoma [87].

In neurosurgery, telemedicine demonstrates its potential with applications such as telehealth stroke triage and ML-based prognostics for neuro-trauma [88]. Recently, a pediatric neurosurgery team utilized ML to screen for craniosynostosis by analyzing digital photographs using semiautomated image analysis software [89]. The ML algorithms accurately identified common types of craniosynostosis, highlighting the effectiveness of this AI-augmented telemedicine approach. This innovative method enhances access to screening for head shape abnormalities, enables early diagnosis, and reduces the burden of ambulatory appointments for families. Primary care providers and non-specialists can also use this approach to identify patients who may require surgical referral [90].

In diabetes management, several projects have emerged utilizing new information and communication technologies and Web 2.0 technologies for automatic data transmission and remote interpretation of patient information [91-93]. A notable aspect of these telemedicine projects is the widespread incorporation of ML, which significantly enhances their capabilities and effectiveness [91-93]. AI algorithms enable more efficient and accurate data analysis, empowering healthcare professionals to make informed decisions and provide personalized care to diabetes patients.

Artificial intelligence-driven behavioral health interventions

Expanding access to digital mental health therapies, the increasing need for psychological services, along with the development of AI, has led to the rise of digital mental health interventions (DMHsI) in recent years. Patient evaluation, symptom evaluation, modifying behaviors, and information dissemination are just a few of the areas where AI-powered chatbots are currently being employed in DMHIs. Chatbots may be as basic as algorithms based on rules such as ELIZA or as complex as AI models that use ML and language processing methods [94].

Support, screening, training, intervention, surveillance, and avoiding recurrence are just a few of the chatbot-based services offered inside DMHIs. Using chatbots for mental health diagnosis and triage might reduce the burden on healthcare workers and expedite the treatment of individuals who need it the most [95]. Chatbots have been used to test for and diagnose a wide range of mental health conditions, including dementia, drug addiction, stress, depression, attempted suicide, anxiety disorders, and symptoms of post-traumatic stress disorder. In this context, the chatbot is used as a conversational counterpart. After the user enters the symptoms they are experiencing into the chatbot, it gives them a medical diagnosis, prognosis, and therapeutic plan (as well as information concerning the diagnosis) [96-105].

Overall, 51% of mental health professionals surveyed thought using chatbots for diagnostic purposes was a challenge. AI-assisted diagnostics, on the other hand, might aid in the early identification of those at risk, allowing for more effective intervention and problem avoidance [106,107]. To properly predict the onset of certain mental health diseases, other kinds of AI may also be able to analyze trends in behavior and information supplied by the person. For instance, ML algorithms possess a 79% success rate in predicting the start of psychosis and a success rate of 96% in identifying the first signs of attention-deficit disorder (ADD)/ADHD and ASD [108].

For instance, certain chatbots leveraging NLP in nature may mimic a therapeutic conversational approach for the purpose of deploying and educating users about different therapies [109]. The most studied and widely used kind of chatbot is one based on cognitive behavioral therapy (CBT), as shown by a meta-analysis that found 10 out of 17 chatbots primarily used CBT [110]. One such chatbot, Woebot, employs NLP to mimic real physicians and social discourse to give CBT to customers through instant messaging [111]. Happify Health, an ad-funded digital mental health provider, added a chatbot this year. Anna is a chatbot powered by AI that simulates a therapist’s job and provides several Happify activities. Anna was designed to work in tandem with other aspects of the Happify program, as opposed to some of the other DMHIs chatbots which operate as separate applications (i.e., the chatbot itself is the intervention). Happify was designed to disseminate enhanced forms of evidence-based activities culled from many therapeutic modalities including CBT, stress reduction using mindfulness, and positive psychology. The activities are divided into *tracks* to assist users in zeroing in on a specific issue, such as stress management [112].

Anna may be found in these tracks. After selecting a route that includes Anna, users are welcomed by the chatbot with a personalized video message and explanation of the chatbot’s capabilities. Listeners may also be polled by Anna with specific questions aimed at collecting information for refining the personalized playlist. To increase user involvement and the likelihood that they will complete the activities as intended, Anna also provides particular assignments inside these tracks. So, Anna helps make the most of the benefits of that move. Anna tracks the elements that contribute most to successful outcomes for each activity, evaluates responses based on those elements, and then prompts participants to fill in any gaps in their initial remarks. Whenever a user’s first response to a task requiring gratitude does not demonstrate an adequate amount of gratitude, Anna will ask for clarification. Anna guides users on how to make better use of the platform and listens to their feedback to allay concerns that conversations with chatbots are not engaging enough [113].

Artificial intelligence in medical education and training

With the increased integration of AI in healthcare, electronic health records (EHRs) can be used for novel techniques such as data processing and enhanced decision-making, prompting accurate data input into EHRs by physicians to maximize the benefits of AI [114]. These shifts into the AI era can positively impact clinical practice and patient health outcomes by training healthcare personnel with better knowledge and skills [115,116]. A single error in AI can cause harm to thousands of patients with wide usage, highlighting the importance of accurate data extraction and input by clinicians to train the AI sufficiently [117]. Therefore, future physicians must understand data management and AI applications. Medical education should incorporate topics about AI, data science, EHR basics, and AI-related ethics into students’ and physicians’ curricula, progressively educating them in their academic journey [118]. Medical schools and healthcare organizations should invest in infrastructure and technological support and collaborate with AI regulatory institutions to efficiently utilize AI [119].

A scoping review by Nagi et al. on current applications of different AI methods such as ML, robotics training, and virtual reality (VR) in several domains of medical education has shown enhanced practical skills of medical students following the implementation of AI-based assistance. Through VR, students can train their decision-making skills in surgical and medical procedures in a controlled and safe manner [116]. Despite the investment requirement in costly hardware infrastructure, the training is still cost-effective compared to traditional manikin/actor-based training in the long run [120]. AI can significantly benefit microsurgical education and ophthalmic surgeon training simulations and video content analytics in a low-risk manner. VR head-mount displays offer several possibilities for operating room simulation [121].

Creutzfeldt et al. trained 12 Swedish medical students in cardiopulmonary resuscitation by avatars to better understand their reactions and experiences using a multiplayer virtual world. The positive aspects of learning cardiopulmonary resuscitation were confirmed after a data-driven approach in qualitative methodology. Further clinical performance should be analyzed to rule out erroneous self-belief bias. Participants noted insufficient psychomotor skills and lack of stress to be unrealistic, which could affect their real-world performance [122].

A meta-analysis by Zhang et al. showed that AI could also be applied in medical education at different stages, such as teaching implementation, evaluation, and feedback. The quality of teaching can be assessed with feedback and evaluation from AI. However, it is challenging to verify the effectiveness of AI implementation [123]. Teachers must be appropriately trained to utilize AI in teaching [124]. It is essential to review the ability of AI compared to physicians with expertise in making healthcare-related decisions. Chat Generative Pre-trained Transformer (ChatGPT), a server-contained language model, was evaluated with 350 US Medical Licensing Exam (USMLE) sample test questions without virtual assets. The questions were formatted and input in the sequence of open-ended prompting, multiple-choice single answer without and with forced justification prompting with a new chat session for every entry to eliminate memory retention bias. ChatGPT scored passing performance for all three steps of USMLE with high internal concordance and significant insight in explanations. The study concluded that large language models have significant potential to assist medical education and clinical decision-making [125].

Although ChatGPT has revolutionizing potential in medical education, it cannot completely replace hands-on clinical experience and mentorship. Moreover, algorithms use large databases, which may contain biases resulting in biased system output that could lead to a loss of fairness in treating minority or underrepresented groups. Therefore, AI systems output should be monitored to ensure the absence of bias and eliminate any biases present [126]. Further research can help with the issue of bias considerably [127].

The use of AI is not without its challenges, such as black box problems [128], data privacy, and liability issues [129]. Hence, physicians should know about the strengths, limitations, and appropriate usage of AI tools [130,131].

Artificial intelligence ethical considerations

Healthcare is a high-risk field in which mistakes can lead to severe consequences for the patient. Patients meet with physicians at a time when their overall health is already compromised, leaving them vulnerable [132]. Therefore, the use of AI in healthcare must undergo rigorous testing and study before becoming a significant part of clinical management. The four primary ethical issues concerning AI in medicine are safety and transparency, algorithmic fairness and biases, informed consent for the use of personal data and health records, and data privacy [124].

The main legal concern is the limited transparency of algorithms used in AI, referred to as the *black box* issue, where the algorithms responsible for the outputs are not disclosed [133,134]. Transparency, particularly accessibility and comprehensibility in AI design and governance, provides vital information that should be attainable and understandable. However, details about the functionality of these algorithms are often intentionally made difficult to obtain [135]. Successful scrutiny of results or outputs obtained from AI systems is hindered as the thinking process that led to them is concealed by modern computing approaches.

The FDA has already approved autonomous AI diagnostic systems based on ML. These ML-healthcare applications (ML-HCAs) create algorithms from large datasets and make predictions without requiring explicit programming to avoid biases and errors [133]. While this new technology has the potential to revolutionize healthcare, it must be properly designed with effective safety measures to ensure data privacy and prevent inaccuracies and breaches [134].

Privacy protection poses challenges to ML. ML necessitates large datasets for effective training, leading to high accuracy in results. However, when significant amounts of data are used to train ML applications from multiple sources, the data’s origin can ultimately be traced back to the patients, negating attempts at privacy [134]. Ownership and rights to datasets containing patient information are complex and vary by jurisdiction and the degree to which data has been de-identified or anonymized [136,137].

In Europe, patients own and have usage rights to all their data, whereas healthcare providers in North America may own the *physical* evidence related to patient data [138,139]. With more protective regulations such as the General Data Protection Regulation (GDPR) [135] in the European Union (EU), patients may be hesitant to allow their data to be used for training AI models. This could lead companies to seek less regulated environments with poorly defined and enforced data ownership regulations. The GDPR might discourage US companies from conducting business due to these restrictions, and EU citizens may not be able to enforce their privacy rights in other jurisdictions [140].

Accountability for AI errors remains a gray area, with no clear individual or entity held responsible. Consequently, AI severely limits the ability to assign blame and/or ownership of the decision-making process [140]. The lack of liability in such a scenario raises concerns regarding professional obligations and patient safety when using unverified AI systems in a clinical setting. To address this, verification and validation are essential when clinicians use AI systems. Managers overseeing AIS users should clarify that physicians cannot avoid accountability by blaming AI systems [132]. Automation bias can occur when a mostly accurate AI system leads medical practitioners to become complacent, causing individuals, both patients and doctors, to accept the system’s results without questioning its limitations. A study by Mirsky and colleagues demonstrated how malware and AI DL tools could mislead expert radiologists about the presence or absence of malignant lung lesions on CT scans [141].

In the study conducted by Buolamwini and Gebru, selection bias was evident in automated facial recognition and the associated datasets, resulting in reduced accuracy in recognizing darker-skinned individuals, particularly women. The datasets used by MLs are derived from specific populations. Consequently, when these AI systems are applied to underserved or underrepresented patient groups, they are more likely to produce inaccurate results [142]. Due to the availability of extensive datasets focusing on common diseases, AI might overlook rare or uncommon conditions that a radiologist could diagnose on X-rays or CT scans [143]. Using models based on training data that do not adequately represent the population, case mix, modalities, and acquisition protocols can cast doubt on the performance and confidence in its use [144].

The contentious debate surrounding the legality of AI systems has prompted governing bodies such as the European Parliament to pass the GDPR [135], aimed at proactively addressing morally challenging situations that may arise from implementing AI in healthcare [145,146]. Integrating AI into medical practice [147-149] requires a well-established governance framework that safeguards patients from harm [150,151]. AI must always adhere to ethical principles to ensure the well-being of patients and should adapt to continuously changing environments with frequent disruptions [151]. As AI becomes more integral to healthcare delivery, AIS and ML-HCAs must meet all ethical requirements and be free from unjust biases. Eventually, AI is likely to surpass humans in some healthcare fields, and refraining from its use would be both unethical and unscientific [152].

Challenges and future directions

AI is a rapidly evolving technical discipline that plays a crucial role in medicine. However, the implementation of AI faces several challenges, including technical, ethical, safety, and financial concerns. AI technology offers significant opportunities for diagnostic and treatment purposes in medicine, but it requires overcoming challenges, particularly related to safety [153]. One of the most significant challenges in medical AI applications, especially in computational pathology, is the lack of labeled data, which is exacerbated by the multi-gigapixel nature of images and high data heterogeneity [154].

The integration of AI into cancer research shows promising outcomes and is currently addressing challenges where medical experts struggle to control and cure cancer. AI provides tools and platforms that enhance our understanding and approach to tackling this life-threatening disease. AI-based systems aid pathologists in diagnosing cancer more accurately and consistently, thereby reducing error rates [155]. AI has also demonstrated high effectiveness in various aspects of drug design and development. However, significant challenges persist, including the acquisition of high-quality data [156].

Despite the considerable advantages and notable progress in contemporary methodologies, rule-based systems commonly employed in medical AI exhibit limitations. These encompass substantial expenses associated with development, constraints arising from the intricate representation of multifaceted connections, and the need for extensive medical expertise [155].

While AI’s involvement in direct patient care is currently limited, its expanding role in complex clinical decision-making processes is on the horizon. Establishing fundamental guidelines for AI’s scope, transparent communication with patients for informed consent, and comprehensive evaluation of AI’s implementation are crucial steps toward setting a universal standard. This emphasizes the importance of algorithmic transparency, privacy protection, stakeholder interests, and cybersecurity to mitigate potential vulnerabilities. Proactive leadership from professional organizations can play a vital role in building public trust in the safety and efficacy of medical AI, thereby driving further advancements in this promising domain [157].

AI has the potential to collaborate with other digital innovations such as telemedicine, enabling virtual consultations and the implementation of the Internet of Medical Things to enhance referral procedures. AI’s capabilities extend to precise risk stratification of cancer stages and the selection of suitable treatment paths. Shared factors are evident across multiple tiers during data analysis, enabling AI models to identify causal links between variables. By amalgamating this knowledge, AI research can achieve higher precision in examining cancer-related medical incidents. In the future, comprehensive databases will emerge, generating new data sets encompassing every aspect of human health. These repositories will empower highly intricate models, capable of personalizing therapy choices, precise dosage calculations, surveillance strategies, timetables, and other pertinent factors [155].

## Conclusions

Prominent AI technologies such as ML and DL have immensely influenced diagnostics, patient monitoring, novel pharmaceutical discoveries, drug development, and telemedicine. Significant innovations and improvements in disease identification and early intervention have been made using AI-generated algorithms for clinical decision support systems and disease prediction models. AI has changed the face of healthcare through unique advancements in patient monitoring, diagnosis, and treatment planning. AI has made strides in optimally implementing clinical drug trials, telemedicine, and personalized therapeutic regimes. However, ethical issues regarding data protection and transparency of AI-driven algorithms remain a cause for concern. Although AI holds promise in mental health interventions, medical education, and virtual training, its role must be aligned with human expertise. The main challenge is to optimize AI’s transformative impact while keeping ethical and regulatory principles in line.
